# Anticoagulant Effect and Platelet Bioactivities of a Novel Cephalopod Byproduct Oil

**DOI:** 10.3390/md24050150

**Published:** 2026-04-23

**Authors:** Ioannis Tsamesidis, Paraskevi Tzika, Diana Samara, Sakshi Hans, Ioannis Zabetakis, Eleni P. Kalogianni

**Affiliations:** 1 Oils and Interfaces Group, Department of Food Science and Technology, International Hellenic University, P.O. Box 141, 57400 Thessaloniki, Greece; johntsame@gmail.com (I.T.);; 2 Division of Transfusion, General Hospital of Naousa, 59200 Naousa, Greece; 3 Department of Chemical Sciences, University of Limerick, V94 T9PX Limerick, Irelandioannis.zabetakis@ul.ie (I.Z.); 4 The Bernal Institute, University of Limerick, V94 T9PX Limerick, Ireland; 5 Health Research Institute, University of Limerick, V94 T9PX Limerick, Ireland

**Keywords:** anticoagulant effect, hemocompatibility, *Nototodarus sloani*, marine oils, oil extraction methods, platelets, byproducts valorization

## Abstract

This study investigates the effects of a novel marine byproduct oil extracted from the cephalopod *Nototodarus sloani* (Arrow squid) on human platelets and red blood cells (RBCs). The oil was produced using enzyme-assisted extraction under varying pH conditions without further refining. The level of oxidation of the different oils was determined. Hemocompatibility and oxidative effects were evaluated after 24 h of incubation at physiological and fever-like conditions. Hemolysis levels varied with extraction conditions and with the amount of oil in contact with the cells. Oils extracted using 0.5% Alcalase^®^ and 1% Protamex^TM ®^ at pH 5.9 demonstrated superior hemocompatibility. Intracellular reactive oxygen species (ROS) levels presented a dose-dependent increase, with higher levels observed in oils extracted at a higher pH. Although there was no direct correlation between hemolysis rate, ROS levels and oxidation, the less oxidized oils presented lower ROS formation and better hemocompatibility. Additionally, the oils exhibited a strong anticoagulant effect and low IC_50_ values against TRAP-6-induced platelet aggregation. These findings highlight the potential of *Nototodarus sloani* as a source of bioactive compounds, providing initial evidence of potential cardiovascular benefits and resource valorization, underlining the importance of extraction conditions in determining the biological properties of marine byproduct oils.

## 1. Introduction

Over the past decade, the food industry has increasingly shifted towards sustainable and health-oriented solutions, with particular emphasis on the use of byproducts as alternative sources of high-value compounds [1]. In particular, the valorization of marine byproducts has emerged as a critical area of innovation [2]. These underutilized resources offer a rich reservoir of bioactive compounds with demonstrated potential to enhance human health, particularly in the prevention and management of chronic diseases [3]. Technologies such as enzyme-assisted extraction enable the efficient recovery of high-value lipids from seafood waste streams [4], aligning with circular economy principles while supporting the development of next-generation functional foods and nutraceuticals. This intersection of sustainability, food science, and human health defines a new frontier in food research. Lately, the effect of edible oils on human health has been examined and beneficial effects of several oils have been identified [5]. Among them is the effect of virgin olive oil on parameters related to inflammation and cardiovascular disease [6,7,8,9]. Another category of oils presenting beneficial effects is marine oils rich in eicosapentaenoic acid (EPA) and docosahexaenoic acid (DHA), which are long-chain omega-3 fatty acids known for their anti-inflammatory, cardioprotective, and neuroprotective properties [10,11,12]. The oxidative status of oils plays a significant role in their health effects, with highly oxidized oils presenting adverse effects [13]. Nevertheless, the literature review shows that our knowledge and comprehension of the effects of edible oils on cells is limited [14]. Furthermore, world production is mainly based on fish byproducts, whereas cephalopods have not been commercially used so far for the production of fish oils [15]. In addition, our knowledge of cephalopod byproduct oil composition, production and its effects on our health is extremely limited [16,17,18].

The European Union’s Landing Obligation (LO) policy has further highlighted the importance of utilizing such byproducts to reduce discards and promote sustainability in fisheries [19]. While extensive studies have evaluated the LO’s effectiveness in minimizing discards, the outcomes vary considerably across different fisheries and geographical regions, underscoring the need for tailored approaches [20]. *Nototodarus sloani* (Arrow squid) inhabits the Pacific and Atlantic oceans [21] and is an abundant species with respect to other squids of the same area [22]. *Nototodarus sloani* is traded worldwide and specifically in the southern part of Europe [23]. The abundance of the species in worldwide trade and as a culinary tradition in many countries highlights the economic and nutritional importance of this species within the marine products sector. Importantly, the byproducts of *Nototodarus sloani* have been identified as a promising source of lipids with high nutritional value, including bioactive compounds that may have applications in human health due to their composition in fatty acids [17] and antioxidant astaxanthin [18]. Despite this potential, the comprehensive utilization of these byproducts for human consumption remains underexplored. Advancements in processing techniques, such as enzyme-assisted extraction, are essential to unlock their full potential and ensure both efficacy and safety in their use.

Cardiovascular diseases (CVDs) remain the leading cause of mortality worldwide, accounting for approximately 17.9 million deaths annually, according to the World Health Organization. Thrombosis, a condition characterized by the formation of blood clots within the vascular system, plays a central role in many life-threatening cardiovascular events such as heart attacks and strokes. Effective treatment and prevention strategies for thrombosis are critical in reducing the global burden of CVDs [24]. Current anti-thrombotic therapies, while effective, are often associated with significant drawbacks, including bleeding risks and the need for long-term management [25]. These challenges highlight the need for innovative therapeutic agents that can mitigate thrombosis without causing adverse side effects. The exploration of bioactive compounds from marine-derived sources, such as those in *Nototodarus sloani* byproducts, may provide new promising avenues for the development of novel treatments. The unique composition of these marine lipids, particularly their omega-3 fatty acids and other bioactive molecules, has shown potential in modulating platelet function, reducing inflammation, and improving vascular health [25,26]. Further investigation into these compounds could contribute to the advancement of safer and more effective anti-thrombotic treatments. The present study aims to investigate the biological effects of oil extracted from *Nototodarus sloani* byproducts on human platelets and red blood cells. Specifically, we assess the impact of oil extracted using enzyme-assisted extraction on the structural and functional integrity of erythrocyte membranes. We examine the hemocompatibility (cytotoxicity) and oxidative effects of this oil after 24 h incubation at physiological (37 °C) and fever temperatures (40 °C). Furthermore, we determine the effect of the extracted oils against TRAP-6-induced aggregation in human-platelet-rich plasma and determine the blood clotting time. These findings will contribute to a deeper comprehension of the bioactive properties of marine-derived byproducts, the effect of extraction variables on these properties, as well as their potential applications in nutrition and health science. The novelty of this work lies in its investigation of a novel oil obtained by valorizing *Nototodarus sloani* byproducts via enzymatically assisted extraction, followed by an in vitro evaluation of their biological functions. It provides, for the first time, new insights into the safety, hemocompatibility, and functional bioactivity of oils derived from this species, highlighting their potential suitability for applications involving direct contact with blood components in circulation.

## 2. Results and Discussion

### 2.1. Oi Composition and Oxidation

As presented previously, *Nototodarus sloani* oil contains a high concentration of omega-3 fatty acids (30.0%) consisting chiefly of EPA (eicosapentaenoic acid) (>10%) and DHA (docosahexaenoic acid) (>10%), independent of the method of extraction; the same holds for the bellies and byproducts of *Tadarodes saggitatus* [16,17]. On the other hand, it is well known that the method of extraction affects the oxidative status of an oil and fish oils are particularly sensitive due to their high concentration of PUFAs [27,28]. Consequently, effects of the extraction method and oxidative status on human erythrocytes and platelets have been identified previously [5]. For this reason, it is considered important to determine the oxidative status of the oils. Also, the oxidative status of the oils affects the perception of rancidity; this is even more so for fish oils, for which secondary oxidation products of ω-3 fatty acids have low molecular weight and low detection thresholds by humans. Therefore, the primary and mainly the secondary oxidation products are related to the rancidity of the oil. Table 1 presents results on the primary and secondary oxidation products (PV and AV respectively) using quality parameters set by the FAO/WHO Codex Alimentarius [29] for the oils extracted from *Nototodarus sloani* byproducts under various enzymatic conditions. The peroxide value (PV), anisidine value (AV), and TOTOX (total oxidation value) are presented. The oils present different oxidative statuses with differences in the indices reflecting the primary value (PV) and secondary oxidation products (AV). For oils extracted using Alcalase^®^ (Sigma-Aldrich Chemie GmbH (Darmstadt, Germany)), the optimal condition was achieved at pH 5.9 with a 0.5% concentration, with the lowest PV, AV and TOTOX values. Increasing the pH resulted in a non-monotonous increase in the PVs and AVs, with the most significant oxidation present at pH 7.5. Substituting Alcalase^®^ with Protamex^TM^ (Novo Nordisk (Bagsvaerd, Denmark)) at the same concentration (0.5%) and acidic conditions (pH 5.9) resulted in increased oxidation of secondary oxidation products. Interestingly, using the Protamex^TM^ enzyme at 1% concentration and pH 5.9 yielded the lowest PV overall (4.5 meqO_2_/kg). Nevertheless, a higher AV (25.3) and TOTOX (34.3) were observed compared to those observed under similar conditions with Alcalase^®^.

### 2.2. Hemocompatibility

**Figure 1:**
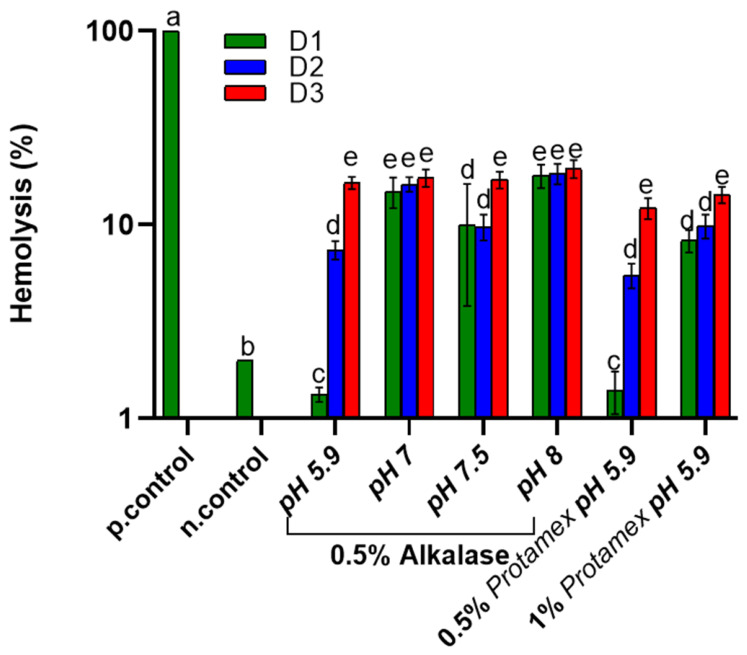
Hemocompatibility of *Nototodarus sloani* oil determined as hemolysis (%) after incubation with healthy erythrocytes at 37 °C. Different letters represent statistically significant differences (*p* < 0.05). D1, D2 and D3 represent different oil doses as defined in the Section 3.

Figure 1 illustrates the hemolysis assay of *Nototodarus sloani* oil byproducts under physiological conditions, determined as the hemolysis rate (%) for three dose rates (D1, D2, and D3).

**Figure 2:**
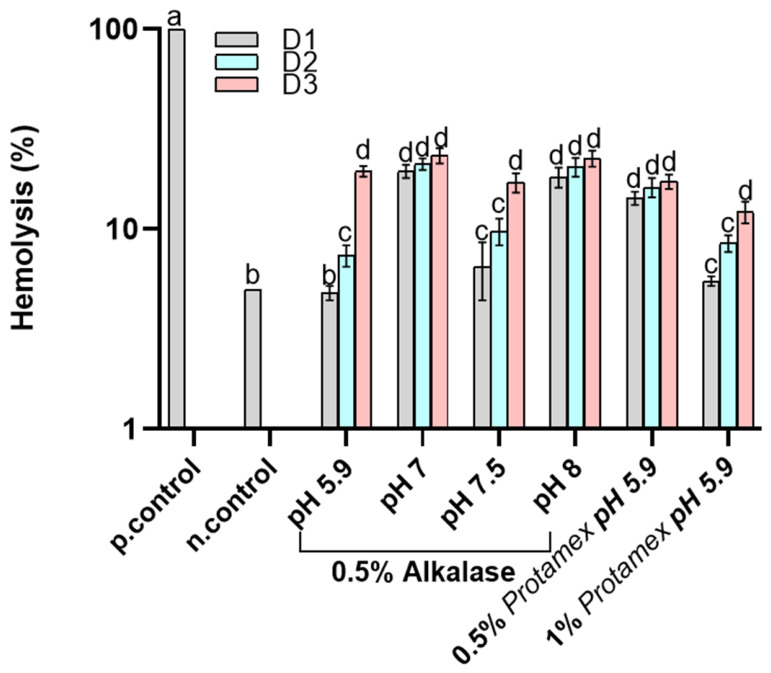
Hemocompatibility of *Nototodarus sloani* oil determined as hemolysis (%) after incubation with healthy erythrocytes at 40 °C. Different letters represent statistically significant differences (*p* < 0.05). D1, D2 and D3 represent different oil doses, as defined in Section 3.

The positive control presents 100% hemolysis, indicating complete lysis of the red blood cells, while the negative control demonstrates minimal hemolysis (2%), confirming excellent hemocompatibility. The lowest hemolysis rate was found for the oil with the lowest oxidation; nevertheless, a correlation between hemolysis and oxidation could not be established since extraction under different conditions is expected to affect the composition of the oils. First, the amount of oil concentration in contact with erythrocytes significantly affected hemolysis in most cases, especially when the oils were enzymatically treated at acidic pH and a low enzyme concentration. In further detail, the oils obtained by both Alcalase^®^- and Protamex^TM^-assisted extraction at low pH (5.9) did not induce hemolysis at the D1 concentration and a dose-dependent hemolysis was observed for higher concentrations at the same pH. Increasing the pH during Alcalase^®^-assisted extraction resulted in an increased hemolysis for all examined oil concentrations (D1-D3). Extracted oil using Protamex^TM^ at 0.5% (*w/w*) and pH 5.9 provided the same results (within statistical variability) for all doserates with Alcalase^®^, verifying the dose-dependent trend for the low pH also for this enzyme. Finally, the increase in Protamex^TM^ concentration resulted in an increase in hemolysis. As denoted above, the results of the hemolysis assay do not correlate with the oil oxidative quality. Enzyme-assisted extraction releases peptides, some of which are oil-soluble and can transfer to the oil phase [30]. Peptides may have adverse effects on the cells, inducing hemolytic activity [31]. This is in line with the increase in hemolysis with a higher enzyme concentration, which would be expected to release more oil-soluble peptides. Figure 2 presents the hemocompatibility of the tested samples under fever-like conditions. The negative control exhibited hemolysis at around 5%, indicating the effects of fever conditions. Besides the effect of fever conditions, which induced an increase in hemolysis in the low-enzyme concentration, low-extraction pH samples as well as the control, the overall trend of the effect of oil on hemolysis is virtually the same both at 37 and 40 °C. Even with some oils showing hemocompatible concentrations, several still caused red blood cell hemolysis. This suggests that hemolysis remains a key limitation of the current formulations. Further refinement is recommended to align the rest of the doses with non-hemolytic levels.

### 2.3. Reactive Oxygen Species

**Figure 3:**
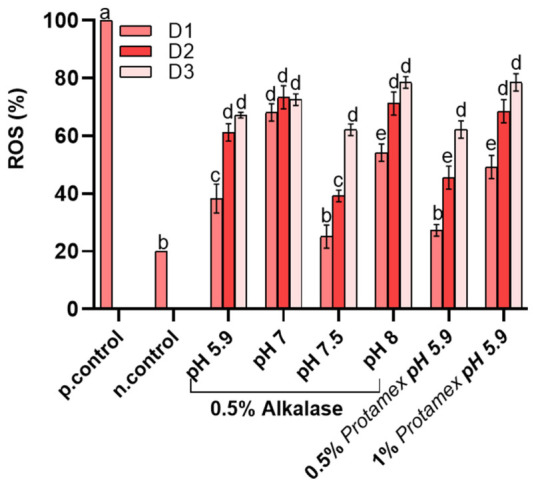
ROS levels of *Nototodarus sloani* oil after incubation with healthy erythrocytes at 37 °C. Different letters represent statistically significant differences (*p* < 0.05). D1, D2 and D3 represent different oil doses as defined in Section 3.

Reactive oxygen species (ROS) are formed by cellular metabolism in living organisms [32]. They can be beneficial to organisms as cell regulators; however, elevated intracellular levels of ROS can lead to “oxidative stress”, which is related to a series of pathologies and aging. The levels of reactive oxygen species (ROS) as a result of the contact of the extracted oils on erythrocytes were determined under various extraction conditions to evaluate oxidative stress, as shown in Figure 3. The positive control (p.control) exhibited the highest ROS levels, with values reaching close to 100% serving as a benchmark for maximal oxidative stress. In contrast, the negative control (n.control) showed basal ROS production. The effect of the oils on ROS formation was dose-dependent, with D1 presenting the lowest ROS generation for each treatment. Among the samples examined, basal ROS levels were obtained for extraction with 0.5% Alcalase^®^ at pH 7 and 0.5% at pH 5.9. On the other hand, the majority of the samples presented higher amounts of ROS, with approximately two- to three-fold increase compared with the negative control. It should be noted that this increase in intracellular ROS can be attributed to the process of erythrocyte senescence. In detail, this increase in ROS can lead to the oxidation of membrane proteins and lipids, causing red blood cells to exhibit signs of “aging,” and macrophages to recognize and remove damaged red blood cells. In inflammatory conditions, erythrocytes can bind to immune complexes via complement receptors (e.g., CR1), transporting them to the liver and spleen for removal and thus playing a passive yet beneficial role in the immune response. Furthermore, in a previous study [13], oxidative stress in red blood cells was linked with the oxidative state of the oil. However, comparing the results of oil oxidation (Table 1) with the results of ROS (Figure 3), no direct relation is found between the primary, secondary oxidation products or the total oil oxidation, as determined by the TOTOX index. Such a result suggests that minor bioactive compounds coextracted in the oil phase may have an effect on the ROS values observed in Figure 3, affecting the physiology of RBCs.

### 2.4. Erythrocyte Membrane Alterations

**Figure 4:**
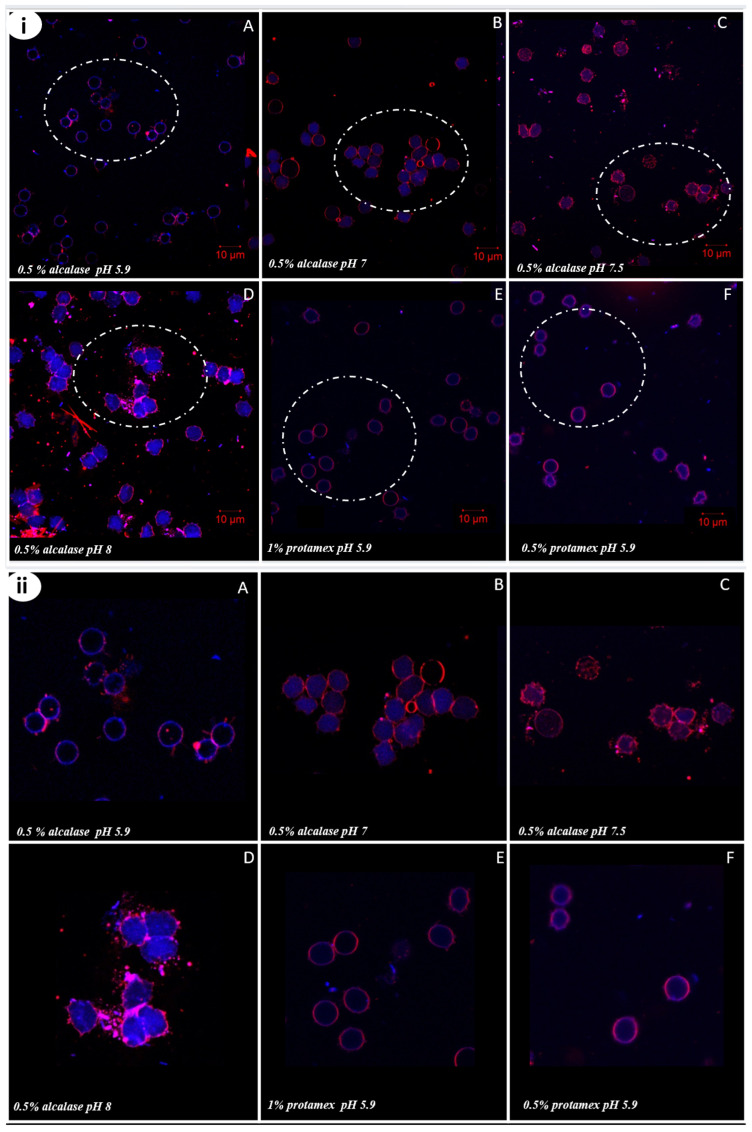
(**i**) CLSM images of erythrocytes in contact with *Nototodarus sloani* oil obtained using different extraction conditions, with dose 1 corresponding to 20 μL of the solubilized oil. (10 μm scale bar). Erythrocytes incubated with oil extracts with Alcalase^®^ (0.5%) at pH 5.9, 7, 7.5, and 8 (**A**–**D**), and Protamex^TM^ (0.5% and 1%) at pH 5.9 (**E**,**F**); the white circle highlights the same erythrocytes at higher magnification, providing a clearer visualization of the alterations in the erythrocyte membrane in (**ii**).

In order to better comprehend the effect of the extracted oils on erythrocytes, the erythrocytes investigated were placed in contact with *Nototodarus sloani* oils, stained with Nile Red and Nile Blue and observed using Confocal Laser Microscopy (CLSM). The results are presented in Figure 4. In detail, Nile Red for the excitation length used specifically stained the membrane phospholipids, while Nile Blue targeted the cytoplasm. This dual staining technique revealed notable alterations in the integrity of the red blood cell membranes. In the Alcalase^®^-treated samples, erythrocyte membrane alterations become increasingly prominent and aggregated with a rise in oil extraction pH. This can be directly correlated to the results on hemocompatibility. On the other hand, in the Protamex^TM^-treated samples at pH 5.9, no significant changes in membrane integrity were observed for both enzyme concentrations used for oil extraction. The observed erythrocyte membrane alterations in all cases align with the hemolysis assay results, confirming that increasing the pH of the oil extraction process leads to a more fragile erythrocyte membrane with noticeable structural alterations. This effect is particularly evident in the Alcalase^®^-treated samples, where higher extraction pH levels correlate with increased membrane disruption and aggregation. The observed erythrocyte membrane alterations in our study align with findings in the literature, indicating that extraction conditions can play a role in oxidative stress and lipid peroxidation, compromising membrane integrity [5,33].

### 2.5. Inhibitory Effect of Nototodarus sloani Oils Towards TRAP-6(Thrombin Receptor Activating Peptide-6)-Induced Aggregation in Human Platelet-Rich Plasma (hPRP)

Atherosclerosis is triggered by the oxidation of cholesterol in the arteries and then activated platelets take part in a domino thrombotic (i.e., aggregation) effect, leading to the formation of atheroma and ultimately atherosclerotic liaison [32]. According to the literature, the IC_50_ (half-maximal inhibitory concentration) values related to the inhibition of platelet aggregation by plant oil extracts typically range from 0.1 to 10 μg/mL, depending on the nature of the extract and the extraction methods used. In particular, extracts that present IC_50_ values below 1 μg/mL are considered particularly effective in inhibiting platelet aggregation, suggesting strong antiplatelet activity, and therefore, strong anti-thrombotic and anti-inflammatory activities [34,35]. Table 2 presents the IC_50_ values of the extracted oils against TRAP-6-induced platelet aggregation. As shown in Table 2, the IC_50_ values (expressed in μg/mL) range from 0.38 to 0.82, and therefore, the results show significant inhibition of platelet activity in human PRP for all examined oil samples. The results show that the oils obtained in this study inhibit platelet aggregation and, in this way, suggest the inhibition of acute inflammation. Thus, they have a strong cardioprotective effect. Statistical analysis using one-way ANOVA revealed statistically significant differences between the oils extracted under different conditions (a = 0.05, *p* = 0.014) and the results on the comparisons of means according to Tukey’s method at the 95% confidence level, as shown in Table 2. Among the samples examined, the best performance was observed for the 0.5% Alcalase^®^-treated samples at pH 8. The results reveal that the enzymatic extraction conditions, particularly enzyme type and pH, influenced the oils’ antiplatelet potency. Marine lipids are rich in polar lipids, which have been linked to their antiplatelet aggregation activity and cardioprotective function [36].

### 2.6. Blood Clotting Time (BCT)

Anticoagulant medications are used to prevent or treat blood clots that can cause strokes, heart attacks, or pulmonary embolisms. Such medicines are heparin, warfarin, and direct oral anticoagulants (DOACs), and are administered at well-established therapeutic doses to achieve precise anticoagulation effects [37]. For example, heparin, commonly used in hospital settings, prolongs clotting time by targeting thrombin and Factor Xa activity [38]. Its therapeutic range is monitored using activated partial thromboplastin time (aPTT), aiming for a 1.5–2.5-fold prolongation of baseline clotting time, translating to approximately 30–70 s. Warfarin, a vitamin K antagonist, achieves anticoagulation by reducing clotting factor synthesis, with its effect monitored through the International Normalized Ratio (INR) to ensure values are maintained between 2.0 and 3.0. Direct oral anticoagulants (DOACs) like dabigatran and rivaroxaban, while requiring less routine monitoring, have well-characterized dose-dependent effects on coagulation assays [39].

**Figure 5:**
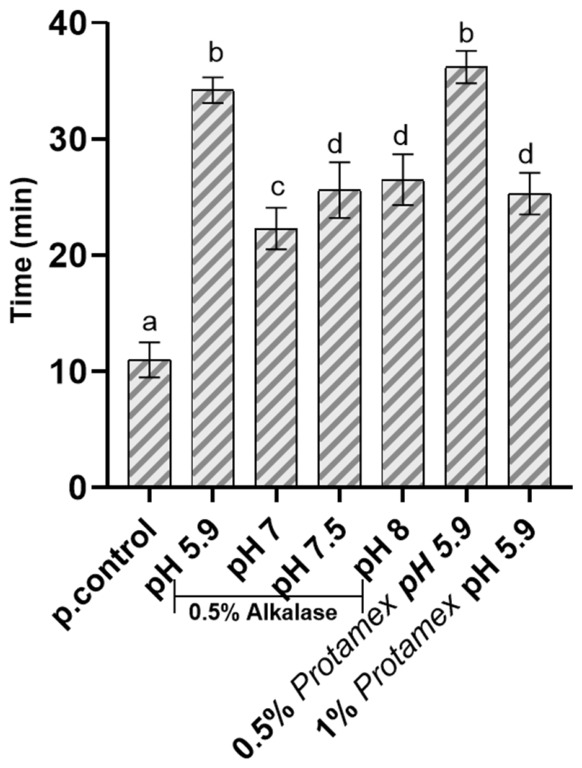
Whole blood clotting time after incubation with the tested oils. Bars indicate the standard deviation of each measurement (*n* = 3). Different letters represent statistically significant differences (*p* < 0.05) (p.control: whole blood treated with CaCl_2_ to induce clot formation).

The effect of the different *Nototodarus sloani* byproduct oils on whole blood clotting time was examined after incubation of the oils with whole blood and compared to the control group (whole blood with the addition of CaCl_2_ to induce the blood clot formation) (Figure 5). The results, as shown in Figure 5, indicate that the oils have a significant effect on clotting time, with all treatment groups showing a prolonged clotting time by at least two-fold, compared to the control group. Also, the inhibition of the coagulation cascade could reduce the generation of proinflammatory mediators (thrombin) and the consequent activation of endothelial and immune cells. Therefore, an anticoagulant agent such as the tested oils may also have anti-inflammatory effects by breaking the feedback loop between coagulation and the inflammatory response.

The above information is especially true for some oil samples (1% Protamex^TM^ -treated and 0.5% Alcalase^®^-treated oil at pH 5.9). In this study, the *Nototodarus sloani* oils exhibited significant clotting time prolongation, with the most potent oils extending normal clotting time to an average of 34 min. This effect surpasses the baseline clotting time of the control group (10 min) by more than threefold, indicating a strong anticoagulant potential. While these results demonstrate efficacy in vitro, it is necessary to evaluate the active components of these oils to determine their pharmacological relevance in comparison to clinical anticoagulants. The anticoagulant effect of Protamex^TM^-extracted oils, particularly at acidic pH, suggests the presence of bioactive compounds that may act through mechanisms similar to or distinct from current anticoagulants. The components of these extracts might interfere with thrombin, Factor Xa, or fibrin polymerization. Further studies should aim to isolate and characterize these components, measure their effective concentrations, and evaluate their potency against standard anti-thrombotic benchmarks. Taking into consideration the pH-dependent effects observed in the erythrocyte membrane from the CLSM analysis, this highlights the importance of optimizing enzymatic extraction conditions to balance yield and functional properties with minimal cytotoxic effects. Furthermore, the observed differences between the lipid fractions obtained using the two commercial proteases may be attributed to variations in their enzymatic specificity and hydrolytic efficiency. Alcalase^®^ is an endopeptidase from *Bacillus licheniformis*, whereas Protamex^TM^ is a mixture of proteases from *Bacillus* sp. Proteases differ in their ability to cleave peptide bonds depending on amino acid sequence and structure, which can lead to differing degrees of deproteination and the generation of residual peptides. Incomplete removal of proteinaceous material may result in the presence of residual proteins or partially hydrolyzed peptides within the lipid fractions. These components could influence the biological responses observed, potentially contributing to the undesirable effects detected in some samples. Overall, our results represent preliminary findings, and further research is needed to confirm the action of compatible oil dosages under clinical conditions. The extraction process and the various factors influencing their activity should be studied in greater depth to optimize and identify the best conditions for enhancing their anticoagulant properties. Additionally, it is essential to isolate and analyze bioactive components to better understand their mechanisms of action in comparison to existing clinical anticoagulants.

## 3. Materials and Methods

### 3.1. Oil Extraction

**Figure 6:**
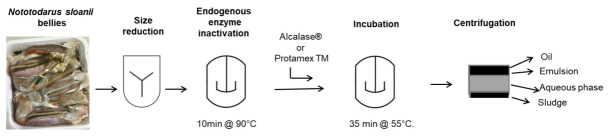
*Nototodarus sloani* flow diagram of extraction process.

The bellies of *Nototodarus sloani* were kindly donated by Atlantida S.A. (Kavala, Greece) and were received frozen (−18 °C) in plastic trays covered with PP plastic film (1 kg of byproduct per tray) and stored at −18 °C. *Nototodarus sloani* was captured at 3–5/2021 in the FAO 81 region. The extraction method was the same as previously described [40] and presented in Figure 6. Briefly, the byproduct was defrosted, chopped and heated at 90 °C for 10 min to inactivate endogenous enzymes. It was then cooled down to 55 °C and enzymes were added at different concentrations (either 0.5% (*w*/*w*) of Alcalase^®^ (Sigma-Aldrich Chemie GmbH company, Darmstadt, Germany) enzyme or 0.5% or 1% (*w*/*w*) Protamex^TM^ enzyme Novo Nordisk (Bagsvaerd, Denmark)) and at different pH levels and subsequently incubated for 35 min under stirring as presented in Table 3. Then enzymes were thermally inactivated and the system was cooled at 4 °C. The oil was obtained by centrifugation. Following separation, the oil was stored at −26 °C in the dark prior to examination. All treatments for enzymes under various conditions were performed in triplicate.

### 3.2. Determination of Oil Oxidation

Oil oxidation is a complex process resulting in a multitude of products. In order to better describe oil oxidation, primary and secondary oxidation products were determined herein using adequate methods for polyunsaturated fatty acids. The determinations described below were used as quality criteria for fish oils. The formation of primary oxidation products (peroxides) of the fish byproduct oil was determined by measuring the peroxide value (PV). For this reason, the method of the European Commission Regulation (EEC) No 2568/91 was followed. The *p*-anisidine value (AV) was used as a representative index of the formation of secondary oxidation products, and the AOCS Official Method Cd 18–9 was used in this case. At least three determinations were performed in each case. Finally, the total oxidation (TOTOX) value was calculated from the above values using the formula TOTOX = 2 PV + AV [41].

### 3.3. Oil Solubilization for Biological Assays

Oil solubilization was carried out using dimethyl sulfoxide (DMSO) in phosphate-buffered saline (PBS). Specifically, 40 μL of the tested oil was mixed with 40 μL of DMSO, and then the solution was diluted with 920 μL of the phosphate-buffered saline (PBS) to create the initial solubilized stock (4% in oil (*v*/*v*)). From this stock, three different doses were prepared: D1 by mixing 20 μL of the solubilized oil with 980 μL of PBS (0.08% in oil (*v*/*v*)); D2 by mixing 40 μL of the solubilized oil with 960 μL of PBS (0.16% in oil (*v*/*v*)); and D3 by mixing 80 μL of the solubilized oil with 920 μL of PBS (0.32% in oil (*v*/*v*)). The hemocompatibility assay was conducted using 20, 25, and 30 μL of the solubilized oil in 980, 975, and 970 μL of erythrocyte suspension (2% hematocrit), respectively. A DMSO control (blank), corresponding to the highest final concentration of DMSO used in the samples, was included in all biological experiments to account for any potential solvent-related effects.

### 3.4. Hemocompatibility Assay

#### 3.4.1. Sample Collection

Red blood cells were isolated from whole blood obtained through the Blood Donation Department of the General Hospital of Naousa, Greece. Donor confidentiality was fully maintained throughout the study. All procedures adhered to Good Clinical Practice guidelines and the principles of the Declaration of Helsinki, in accordance with approval from the hospital’s Ethics Committee (ID_233205920).

#### 3.4.2. Hemolysis Assay

Hemolysis of erythrocytes with the tested oil preparations was evaluated as follows: RBCs were suspended in PBS to obtain a final erythrocyte concentration of 2% (final volume: 1 mL; hematocrit: 2%). The diluted RBCs were then exposed to various concentrations of the preparations, as previously described (oil solubilization for 60 min, followed by incubation for 24 h at 37 °C and 40 °C). The supernatant from untreated RBCs served as the negative control (Ctrl−), while RBCs treated with distilled water were used as the positive control. Hemoglobin absorbance was measured at 541 nm, using 700 nm as the reference wavelength. The percentage of hemolysis was calculated based on at least five independent measurements, as previously described [5].

### 3.5. Fluorescence Analysis for the Detection of ROS Levels

Intracellular reactive oxygen species (ROS) were detected using the cell-permeable probe 2′,7′-dichlorodihydrofluorescein diacetate (CM-H2DCFDA), as previously described (Tsamesidis et al.) [5]. Baseline ROS levels were assessed following incubation of human RBCs (hRBCs) with the tested oil preparations. Oxidation of CM-H_2_DCFDA with fluorescein at 520 nm (λex = 480 nm) (prepared as a 0.5 mM stock solution in DMSO) within RBCs was evaluated by measuring fluorescence in RBC suspensions (0.2% hematocrit) using 96-well black-walled microplates (Corning^®^, Glendale, AZ, USA, catalog number 3340) and a Tecan fluorescence reader. Relative fluorescence was expressed as the percentage of maximal emission, with the positive control (control+) defined as representative of fluorescence intensity obtained after treatment with 3 mM H_2_O_2_. Untreated RBC suspensions served as the negative control. All experiments were conducted in triplicate (*n* = 3).

### 3.6. Confocal Laser Scanning Microscopy (CLSM)

The same experimental procedure conducted for the hemolysis assays was also used to image cells after their interaction with different types of oil from different extraction conditions using dose 1 (D1) corresponding to 20 μL of the solubilized oil. To visualize the erythrocyte membrane, fresh blood samples were collected, washed, and incubated with the six oil types mentioned in the Section 3.4.2. A small droplet (10 μL) of the suspension was stained with Nile Red and Nile Blue dyes and incubated for 10 min. The prepared slide was then examined using a confocal laser scanning microscope (CLSM), model EVO 50XVP (Carl Zeiss, CZ Microscopy GmbH, Jena, Germany). Dual-channel lasers (Ar/K and He/Ne) were used, with Nile Red and Nile Blue excited at wavelengths of 625 nm and 660 nm, respectively. High-resolution images were taken using oil-immersion lenses with magnifications of 40× and 60×.

### 3.7. Inhibitory Effect of Nototodarus sloani Oils Towards TRAP-6-Induced Aggregation in Human Platelet-Rich Plasma (hPRP)

Venous blood was collected from three healthy volunteers (two females and one male), all of whom provided written informed consent prior to participation. Ethical approval for the platelet aggregation studies was granted by the Ethics Committee of the University of Limerick (approval number: 2022_01_01_S&E). The study was conducted in accordance with Good Clinical Practice guidelines and the Declaration of Helsinki. Exclusion criteria, which included the use of medication for cardiovascular conditions, intake of anti-inflammatory drugs, smoking, or the presence of health conditions such as cardiovascular disease or coagulation disorders. Anti-thrombotic activity was assessed using human platelets, as previously described [36].

Blood samples were collected in S-Monovettes and centrifuged at 194× *g* for 18 min at 20 °C without break using an Eppendorf 5702R centrifuge (Eppendorf Ltd., Stevenage, UK). The resulting supernatant, platelet-rich plasma (PRP), was transferred into polypropylene tubes at room temperature for use in aggregation assays. Platelet-poor plasma (PPP) was obtained by further centrifugation at 1465× *g* for 20 min at 20 °C, also without break. Platelet concentration in PRP was adjusted to 500,000 platelets/µL, when necessary, by dilution with PPP, based on absorbance measurements obtained using a Shimadzu UV-1800 spectrophotometer (Kyoto, Japan), prior to analysis with a Chronolog-490 two-channel platelet aggregometer.

TRAP-6 (Merck) was prepared in sterile distilled water at a working concentration of 0.5 mM. Fish oil samples were initially solubilized in DMSO at a 1:2 ratio to ensure complete dissolution. A subsequent dilution (solution B) was prepared in 1× PBS to achieve a final DMSO concentration of 0.1%. A further serial dilution (solution A) was then prepared, resulting in a final DMSO concentration of 0.01%, minimizing its potential effect on platelet inhibition. Solution A was used as the working solution for platelet aggregation analysis.

Platelet aggregation was measured using a Chronolog-490 two-channel turbidimetric aggregometer (Havertown, PA, USA) with AGGRO/LINK 8 software, version 2.0. Briefly, 250 µL of PRP was added to an aggregometer cuvette, maintained at 37 °C and stirred at 1200 rpm. PPP was used as a blank for calibration. TRAP-6 (0.5 mM) was added to induce maximal reversible aggregation in the absence of lipid samples. For each lipid preparation, the amount required to inhibit 50% of TRAP-6-induced aggregation was determined, and IC_50_ values were calculated as previously described [5]. All experiments were performed in triplicate (*n* = 3).

### 3.8. Determination of Blood Clotting Time (BCT)

Blood clotting time (BCT) was determined as previously described [5] using the same oil doses applied in the platelet aggregation assays with hPRP. Briefly, each sample was incubated in Eppendorf tubes at 37 °C in a water bath for 10 min. Coagulation was initiated by adding 20 μL of 0.2 M CaCl_2_ to 340 μL of whole blood, followed by continued incubation at 37 °C.

Clot formation was assessed by inverting the tubes every 10 s; the BCT was defined as the time point at which no flow was observed upon inversion (with tubes held inverted for 1 s before returning to their original position). A control sample without any added materials was included for comparison.

All measurements were independently recorded by three observers. Experiments were conducted in triplicate (*n* = 3), and the resulting IC_50_ values were expressed as the mean lipid mass (µg) present in the aggregometer cuvette ± standard deviation (SD)

### 3.9. Statistical Analysis of Data

For the majority of the in vitro experiments (hemocompatibility, ROS, and BCT), a statistical *t*-test and one-way analysis of variance (ANOVA) were performed via the use of the GraphPad Prism 8.4.2 software program, and the significance level was determined at a = 0.05. For the TRAP-6-induced aggregation assay, one-way ANOVA was also used at a = 0.05, followed by Tukey’s method at a 95% confidence level for multiple comparison analysis. This was performed using Minitab^®^ 22.2.2 (Minitab Inc., State College, PA, USA).

## 4. Conclusions

This study presents for the first time the in vitro effects of a novel squid byproduct oil obtained from enzymatically assisted extraction of *Nototodarus sloani* bellies. It further highlights the significant effect of enzymatic extraction conditions on the oxidative stability, hemocompatibility, bioactivity, and blood clotting properties of *Nototodarus sloani* oil. The oils extracted using Alcalase^®^ at pH 5.9 demonstrated the best oxidative stability, with the lowest peroxide value (PV) and TOTOX, indicating superior quality and reduced oxidative stress. However, the hemocompatibility of the oils varied with pH, showing increased hemolysis at higher pH levels, particularly in Alcalase^®^-treated oils. This suggests that hemolysis is a limitation of using the above oils and that refinement is required. Nevertheless, under fever-like conditions, the oils extracted at a neutral to slightly alkaline pH exhibited stronger hemocompatibility, with elevated levels of ROS at higher pH. In terms of bioactivity, all oils had low IC_50_ values against TRAP-6-induced platelet aggregation. Platelet aggregation is linked to inflammation and atherosclerosis as previously described [42]. Therefore, the strong bioactivities of the extracts against platelet aggregation, as described in this work, may lead to a method being discovered where side streams of aquaculture can be valorized by turning them into significant lipid fractions against inflammation and cardiovascular diseases. In addition, the oils presented a strong anticoagulant effect, with the strongest among them being the oils extracted at pH 5.9 using Protease^®^ at 1% (*w*/*w*) or Alcalase^®^ at 0.5%. Overall, enzymatic extraction conditions, especially enzyme concentration and pH, play a crucial role in determining the oil’s oxidative stability, bioactivity, hemocompatibility, and anticoagulant potential. *Nototodarus sloani* oils extracted at pH 5.9, particularly using Protease^®^ or Alcalase^®^, demonstrated the best bioactivity and clotting time effects, suggesting their potential for further development in biomedical and food applications. Furthermore, the pronounced anticoagulant effects of these oils indicate their promise as nutraceuticals for cardiovascular health, potentially offering natural alternatives or complements to synthetic anticoagulants. Refining the extraction methods further, such as optimizing enzyme concentrations, reaction times, and pH, could enhance the oils’ bioactivity and quality, paving the way for more effective and stable formulations. These findings open new avenues for utilizing *Nototodarus sloani* oils in both functional foods and therapeutic contexts.

## Figures and Tables

**Table 1:** Oil oxidation as described by the peroxide value (PV), anisidine value (AV) and TOTOX values.

Enzyme/Concentration (*w*/*w*)/pH	PV (meqO_2_/kg)	AV (−)	TOTOX (−)
Alcalase^®^ 0.5%, pH 5.9	5.3 ± 0.6	14.2 ± 0.0	24.8
Alcalase^®^ 0.5%, pH 7.0	8.5 ± 0.6	14.7 ± 1.0	31.7
Alcalase^®^ 0.5%, pH 7.5	8.5 ± 0.6	29.2 ± 0.9	46.2
Alcalase^®^ 0.5%, pH 8.0	8.5 ± 0.6	18.8 ± 1.1	35.8
Protamex^TM^ 0.5%, pH 5.9	7.0 ± 0.0	29.2 ± 0.2	43.2
Protamex^TM^ 1.0% pH 5.9	4.5 ± 0.7	25.3 ± 0.3	34.3

**Table 2:** Bioactivity of *Nototodarus sloani* oil expressed as IC_50_ against TRAP-6-induced platelet aggregation; results on the comparisons of means according to Tukey’s method at a 95% confidence level are shown in superscript.

Enzyme, Concentration (*w*/*w*), pH	IC_50_ (μg/mL) (Average ± St. Dev.)
Alcalase^®^, 0.5%, pH 5.9	0.72 ± 0.02 ^ab^
Alcalase^®^, 0.5%, pH 7.0	0.82 ± 0.25 ^a^
Alcalase^®^, 0.5%, pH 7.5	0.68 ± 0.15 ^ab^
Alcalase^®^, 0.5%, pH 8.0	0.38 ± 0.08 ^b^
Protamex^TM^, 0.5%, pH 5.9	0.61 ± 0.07 ^ab^
Protamex^TM^, 1.0% pH 5.9	0.53 ± 0.16 ^b^

**Table 3:** Enzyme-assisted extraction parameters.

Enzyme Type	Concentration (*w*/*w*, %)	pH
Alcalase^®^	0.5	5.9
Alcalase^®^	0.5	7.0
Alcalase^®^	0.5	7.5
Alcalase^®^	0.5	8.0
Protamex^TM^	0.5	5.9
Protamex^TM^	1.0	5.9

## Data Availability

Data will be made available on request.

## Abbreviations

The following abbreviations are used in this manuscript:

Eicosapentaenoic acid (EPA), docosahexaenoic acid (DHA), reactive oxygen species (ROS), cardiovascular diseases (CVDs), World Health Organization (WHO), thrombin receptor activator peptide-6 (TRAP-6), peroxide value (PV), European Commission Regulation (EEC), p-anisidine value (AV), total oxidation (TOTOX), confocal laser scanning microscopy (CLSM), red blood cells (RBCs), dimethyl sulfoxide (DMSO), phosphate buffer saline (PBS), positive control (p.control), negative control (n.control), human platelet-rich plasma (hPRP), dose (D), blood clotting time (BCT).
